# Model-Based Individualized Treatment of Chemotherapeutics: Bayesian Population Modeling and Dose Optimization

**DOI:** 10.1371/journal.pone.0133244

**Published:** 2015-07-30

**Authors:** Devaraj Jayachandran, José Laínez-Aguirre, Ann Rundell, Terry Vik, Robert Hannemann, Gintaras Reklaitis, Doraiswami Ramkrishna

**Affiliations:** 1 School of Chemical Engineering, Purdue University, 480 Stadium Mall Way, West Lafayette, IN, 47907, United States of America; 2 Weldon School of Biomedical Engineering, Purdue University, 206 South Martin Jischke Drive, West Lafayette, IN, 47907, United States of America; 3 Riley Hospital for Children, 702 Barnhill Drive, Indianapolis, IN, 46202, United States of America; University of Erlangen-Nuremberg, GERMANY

## Abstract

6-Mercaptopurine (6-MP) is one of the key drugs in the treatment of many pediatric cancers, auto immune diseases and inflammatory bowel disease. 6-MP is a prodrug, converted to an active metabolite 6-thioguanine nucleotide (6-TGN) through enzymatic reaction involving thiopurine methyltransferase (TPMT). Pharmacogenomic variation observed in the TPMT enzyme produces a significant variation in drug response among the patient population. Despite 6-MP’s widespread use and observed variation in treatment response, efforts at quantitative optimization of dose regimens for individual patients are limited. In addition, research efforts devoted on pharmacogenomics to predict clinical responses are proving far from ideal. In this work, we present a Bayesian population modeling approach to develop a pharmacological model for 6-MP metabolism in humans. In the face of scarcity of data in clinical settings, a global sensitivity analysis based model reduction approach is used to minimize the parameter space. For accurate estimation of sensitive parameters, robust optimal experimental design based on D-optimality criteria was exploited. With the patient-specific model, a model predictive control algorithm is used to optimize the dose scheduling with the objective of maintaining the 6-TGN concentration within its therapeutic window. More importantly, for the first time, we show how the incorporation of information from different levels of biological chain-of response (i.e. gene expression-enzyme phenotype-drug phenotype) plays a critical role in determining the uncertainty in predicting therapeutic target. The model and the control approach can be utilized in the clinical setting to individualize 6-MP dosing based on the patient’s ability to metabolize the drug instead of the traditional *standard*-*dose*-*for*-*all* approach.

## Introduction

Cancer has been a perennial challenge to clinicians and researchers ever since its existence has come to light. Despite the remarkable progress in healthcare in the past few decades, cancer remains the second leading cause of deaths, accounting for about 1 in 4 deaths in the US [1]. One of the primary reasons for the failure of cancer treatment can be attributed to high inter-patient variability in response to such treatment. The existing treatment modalities are “effective” only in subsets of patient population due to a significant *genetic* and *phenotypic* variation among patients. It is this intrinsic variation, even within the same genotypic group, which renders the clinical decision far from straightforward. The dose regimen determined during randomized clinical trials involving a small number of patients may be appropriate at best for an “average” patient because these studies are designed to define the best dose for the whole population, and not for any specific patient. Thus, it produces severe toxicity in some patients and insufficient treatment in others. Adverse drug reactions has been estimated to cause about 2 million hospitalizations and 100,000 deaths per year in the US [2], necessitating a dire need for a rational approach to individualized treatment.

### Challenges of 6-MP Treatment

6-MP is one of the important drugs in a series of purine analogues. In addition to many pediatric cancers, 6-MP is a key drug in inflammatory bowel disease (IBD) and many autoimmune diseases. Common acute side-effects during 6-MP treatment include myelosuppression, pancreatitis, gastrointestinal intolerance and hepatotoxicity. Besides acute side-effects, many clinical studies reported several chronic effects related to 6-MP treatment. For instance, in patients who have undergone 6-MP treatment for acute lymphoblastic leukemia (ALL), recurrent ALL, secondary neoplasm and other multiple chronic medical conditions are prevalent [3,4]. Clinical studies show that inadequate therapy leads to recurrent ALL whereas aggressive treatment results in acute side-effects and secondary malignancies, thus calling for optimization and individualization of 6-MP dosing [4–9].

6-MP undergoes extensive intracellular metabolism to yield 6-thioguanine nucleotide (6-TGN) (active metabolite) and other methylated metabolites of mercaptopurine (MeMP) [10]. 6-TGN and MeMP are catalyzed by enzymes hypoxanthine-guanine phosphoribosyltransferase (HGPRT) and thiopurine methyltransferase (TPMT) respectively. The relative activities of HGPRT and TPMT are genetically transcribed and regulated for a given patient. Among these, TPMT enzyme activity appears to be the rate limiting step and hence dictates the net concentration of 6-TGN [11]. Much of the treatment variability during 6-MP treatment is cascaded down from the genetic polymorphism exhibited in the *TPMT* gene [12]. For instance, in patients with high TPMT activity, the 6-TGN pathway is suppressed, resulting in low 6-TGN concentration and hence treatment failure. On the other hand, in patients with low TPMT activity, the 6-TGN metabolism is elevated which eventually results in life-threatening myelotoxicity. Thus, TPMT genetic polymorphism is highly correlated with treatment outcome [5,13–15]. Hence, the utilization of the TPMT genotype as a pharmacogenetic marker has been suggested to guide the treatment protocol for an individual patient [16].

### Genotyping vs. Phenotyping

Dosing decision for a specific patient can be made with any one of the following biological markers associated with a drug-disease combination: i) DNA sequence, ii) gene expression profile, iii) protein/enzyme concentration/activity, iv) metabolite/drug concentration and v) cell/clinical response. We refer to this as biological chain-of-response. Pharmacogenomics, at the crossroads of genetics and pharmacology, sheds light on the causative genetic variants influencing treatment response under drug intervention. When introduced, pharmacogenomics was believed to alleviate many of the issues arising in current medical sciences, especially personalized treatment [17,18]. However, it has produced limited success in some of the drug-disease applications that were deemed to be classical cases for pharmacogenomics based personalization [19,20]. Although pharmacogenomics provides some information vital for predicting treatment outcome in the subgroups of patient population, its ability to explain within-group variation may be in question. A detailed account this subject can be found in [21].

Pharmacogenomics relies on a static snapshot of a specific DNA sequence or gene expression and assumes a deterministic evolution of biomolecular events resulting in a predictable cellular response for a given gene variant. In addition, it considers each gene as an independent causal factor for the observed response. However, human physiology is a complex dynamic system and cellular response is a manifestation of interplay between many levels of physiological processes and molecular entities. Furthermore, human physiology is complicated by homeostatic feedback loops, molecular crosstalk and bypass mechanisms that can lead to unexpected clinical outcomes. As a result, within a specific genotype, there is a distribution of phenotypes across patient population. For example, in the case of 6-MP, although there are only a few validated TPMT genotypes, there are as many enzyme activity levels as there are patients. To make things even more complex, within a specific enzyme activity range, there are as many active drug concentrations (the ultimate manifestation of the genotype) as there are patients. Hence, 6-TGN concentration, which is responsible for the cytotoxicity, should be regarded as the ultimate covariate for dose individualization.

### Importance of Individualized Treatment

The foregoing perspective is more clearly evident from a case-study on a TPMT deficient patient who had undergone 6-MP treatment [22]. As soon as the treatment was initiated, the patient’s vital cellular counts plummeted below the critical level. Following several treatment interruptions and, blood/platelet transfusions due to severe myelotoxicity, TPMT genotype and phenotype were assayed at week 20; 6-TGN concentration was also measured. Even with this information, it took another 45 weeks to arrive at the optimal dose through *trial-and-error* with additional treatment interruption and blood/platelet transfusions. It is clear that the current practice of patient titration and qualitative use of vital information is severely deficient. This trial-and-error process has a profound impact on immediate and long-term patient health and hence on allocation of limited clinical resources.

**Fig 1:**
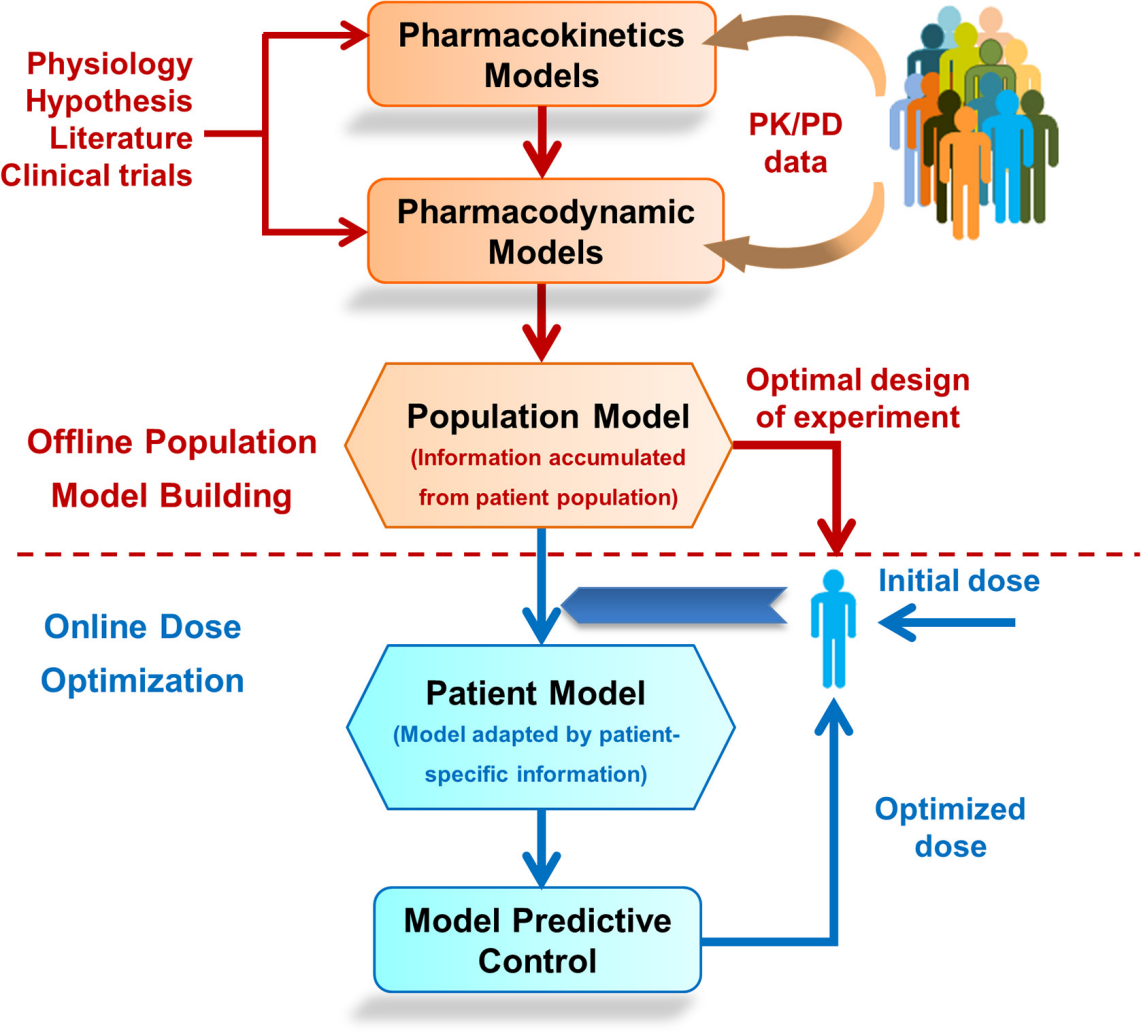
A general framework for model-based individualized dosing of chemotherapeutic drugs. Pharmacokinetic and pharmacodynamic models are formulated based on underlying physiology. With extensive data from a large cohort of patients, a population model is formulated based on the Bayesian approach. A few measurements from a new patient, collected at optimal time points, enables the adaptation of the population model to an individual behavior. Patient models are used to optimize the dose based on model predictive control to maintain the drug concentration within the therapeutic window. In this work, only pharmacokinetic aspects of 6-MP are considered.

There is a growing body of literature that acknowledges this state of affairs and suggests the need for tailoring the dose regimen based on a patient’s genetic and phenotypic make-up [8,23–25]. Given the dynamic nature of physiological responses, it has to be an ongoing process rather than a ‘study-and-adopt’ approach. In other words, following a detailed analysis and accumulation of information during the study phase, a minimum of information must be obtained from each new patient to adapt the approach to the patient before making predictions and dose optimization. Given the significant limitation on continuous monitoring in the clinical settings, a robust model-based *in silico* approach, adaptable to individual patients, is indispensable. A recent report by the National Academy of Engineering and the Institute of Medicine highlights the potential of such engineering approaches, consummated through a partnership between healthcare professionals and engineers, in patient focused health care delivery [25]. Hence, these factors form the thrust of this manuscript and are summarized in Fig 1. In section 2, we describe the model and methodologies used and provide some important results in section 3. Finally in section 4, we conclude with discussion on the impact, constraints in clinical implementation and possible extension.

## Materials and Methods

### Problem Statement

A generic dynamic model for any given drug and patient can be described by a system of ordinary differential equations of the following form,

$$\begin{array}{l} \frac{dx_{i}\left(t\right)}{dt}=X\left(x_{i}\left(t\right),\theta_{i},u_{i}\left(t\right)\right);\;x_{i}\left(0\right)=c\\ \;{\hat{y}}_{i}(t)=Y\left(x_{i}\left(t\right),\theta_{i},u_{i}\left(t\right)\right) \end{array}$$ (1)

where **x**_*i*_(*t*) ∈ ℜ^*n*^: state variables, **θ**_*i*_ ∈ ℜ^*p*^: model parameters, **u**_*i*_(*t*) ∈ ℜ^*q*^: drug input, **c** ∈ ℜ^*n*^: initial conditions, ${\hat{y}}_{i}\left(t\right)\in {ℜ}^{l}\;:$ predicted model output. Patient population is characterized by the random parameter matrix **Θ** ∈ ℜ^*p*^ × ℜ^*N*^ so that an individual patient, *i*, is represented by a vector **θ**_*i*_. Further, we assume that the model parameters **θ**_*i*_ can be partitioned into highly sensitive parameters ${\hat{\theta }}_{i}\in {ℜ}^{s}$ and less sensitive parameters ${\tilde{\theta }}_{i}\in {ℜ}^{r}={ℜ}^{p-s}$ such that,

$$\theta_{i}=\left[\begin{array}{c} {\hat{\theta }}_{i}\\ {\tilde{\theta }}_{i} \end{array}\right]$$ (2)

Sensitive parameters are used to identify patient specific parameters whereas the rest are fixed at population means. Let *y*_*ij*_ denote the *j* ^*th*^ measurement for the *i* ^*th*^ patient, at time *t*_*ij*_. It is not uncommon for these individual measurements *y*_*ij*_ to be corrupted by measurement and assay error, besides model misspecification. If we assume that the modeling and experimental errors are additive in nature, then the observed concentration would be given by,

$$y_{ij}\left(t_{ij}\right)={\hat{y}}_{ij}\left(\theta ,t_{ij}\right)+\varepsilon_{i}\left(x_{ij},u_{ij},\xi_{ij}\right),\;i=1,\ldots ,N;\;j=1,\ldots ,M$$ (3)

For practical purposes, the errors can be assumed to be independent and normally distributed

$$\varepsilon_{ij}\approx N\left(\mu ,\sigma_{ij}^{2}\left(x_{ij},u_{ij},\xi_{ij}\right)\right)$$ (4)

where *μ* = *E*[*ε*_*ij*_ (*t*_*ij*_)] = 0 and *σ*_*ij*_^2^ (*x*_*ij*_, *u*_*ij*_, *ξ*_*ij*_) = *Var*[*ε*_*ij*_ (*t*_*ij*_)]. *ξ*_*ij*_ represents error model parameters. Unlike chemical systems and some biological systems, where the model parameters are more or less constant once the experimental conditions are fixed, human physiology is subject to high inter-subject variability. In other words, for a given disease and drug dosing, no two patients will respond in exactly the same way. As such, although the model structure can be assumed to be identical for individual patients, the model parameters vary significantly among patients. For the *i* ^*th*^ patient, the model parameters are given as,

$$\theta_{i}=\bar{\theta }+\eta_{i}$$ (5)

where $\bar{\theta }$ is the typical value of model parameters (population mean) and **η**_**i**_ ∈ ℜ^*p*^ are independent vectors representing the deviation of the *i* ^th^ patient’s parameters from the population mean values. From Eq (5), it is clear that an individual patient is a part of the population described by a multivariate probability distribution,

$$P\left(\theta_{i}\right)=M_{p}\left(\bar{\theta },\Sigma \right)$$ (6)

In the above equation, $M_{p}\left(.,.\right)$ represents *p*-dimensional multivariate distribution and Σ ∈ ℜ^*p*^ × ℜ^*p*^ is an inter-patient variance-covariance matrix. Much of the challenge in population modeling and dose individualization resides in robust estimation of $P\left(\theta_{i}\right)$ for a new patient within physiological and clinical constraints. A brief description of various methods available to determine $P\left(\theta_{i}\right)$ may be found in [24]. In this work, we are exploiting the Bayesian approach which is elaborated in [24,26,27]. Under the Bayesian framework, several steps are included: $M_{p}\left(\bar{\theta },\Sigma \right)$ is evaluated off-line with the availability of drug dose and drug concentration data from a large number of patients. Based on the variability in response propagated by $M_{p}\left(\bar{\theta },\Sigma \right)$, optimal sampling times that minimize the uncertainty in parameter estimation are estimated. For each new patient, given a measurement at an optimal time, $P\left(\theta_{i}\right)$ and optimum dosing profiles are determined on-line.

### Experimental Data

The data for characterizing 6-MP metabolism and cellular 6-TGN concentration were obtained from three separate clinical studies reported in the literature. In the first study (*D1*), the 6-TGN concentration was measured in a group of 23 patients undergoing 6-MP treatment [28]. Up to four data points were collected per patients over a period of eight weeks. The second set of data (*D2*) for 6-TGN concentration was collected from patients undergoing 6-MP treatment under “per protocol” group in [29,30]. 6-TGN concentration was measured from eight patients over 20 weeks. A third set of data (*D3*) was collected from a clinical study on ALL patients in which 102 measurements of TPMT enzyme activity and corresponding 6-TGN concentrations were collected [31]. This data set is primarily used to determine the dosing profile at the beginning of the treatment when 6-TGN measurement is not available and hence the dosing decision has to be made with TPMT enzyme activity measured at *t* = 0. *D3* is also extensively used for evaluating uncertainty in phenotype prediction with various data along the chain-of-response. The data sets that showed a clear indication of treatment discontinuation or significant dose reduction were eliminated.

### Modeling 6-MP Metabolism

**Fig 2:**
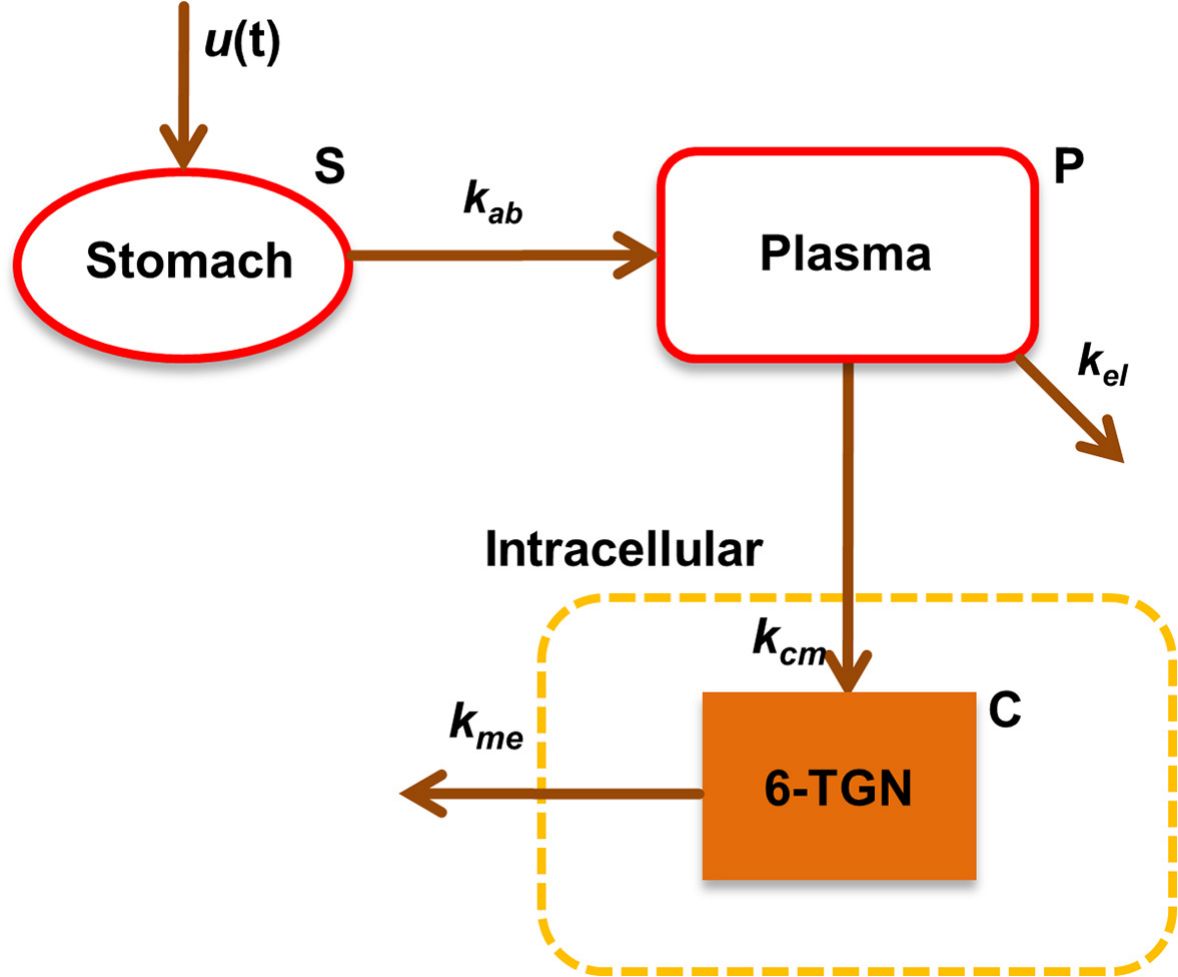
Schematic representation of 6-MP metabolism. The model equations for 6-MP metabolism based on mass-action kinetics are shown in Eq (7).

Although the TPMT pharmacogenomics and the metabolism of 6-MP is one of the extensively studied systems in clinical pharmacology literature, utilization of quantitative dose optimization strategies are limited. Hawwa et al. [32] performed a population pharmacokinetics study of 6-MP in pediatric patients considering TPMT *genotype* as one of the main covariates to characterize inter-patient variability. Phenotypic variations and/or dose optimization strategies were not considered in their work. A simplified schematic of the 6-MP intracellular metabolism accounting for 6-TGN production is shown in Fig 2. Following oral intake to the gut, 6-MP is absorbed at the rate of *k*_*ab*_ into the plasma where it undergoes extensive hepatic clearance at the rate of *k*_*el*_. From the plasma, 6-MP is transported into the intracellular space where it undergoes metabolic conversion. Since negligible intracellular concentration of 6-MP has been reported [33], we assume that 6-MP is metabolized as soon as it enters the intracellular space. The desired pathway leading to 6-TGN is initially catalyzed by HGPRT, followed by a series of other metabolic conversions at a lumped rate of *k*_*cm*_. 6-TGN is eliminated from the cells at a constant rate of *k*_*me*_.

$$\begin{array}{l} \frac{dx_{g}}{dt}=-k_{ab}x_{g}+d(t)\\ \frac{dx_{c}}{dt}=k_{ab}x_{g}-k_{el}x_{c}-\frac{k_{cm}x_{c}}{K+x_{c}}\\ \frac{dx_{m}}{dt}=\frac{\nu_{cm}k_{cm}x_{c}}{K+x_{c}}-k_{me}x_{m} \end{array}$$ (7)

The state variables are defined as follows: *x*_*g*_, amount of 6-MP in the gut (picomole (pmol)); *x*_*c*_, amount of 6-MP in the plasma (pmol); *x*_*m*_, concentration of metabolite 6-TGN in peripheral RBCs (pmol/8x10^8^ RBCs). In the above model, *x*_*m*_ is the observed variable. Conversion of 6-MP into 6-TGN follows *Michaelis*–*Menten* kinetics with reaction rate *k*_*cm*_ and *Michaelis*–*Menten* constant *K*. Patient specific TPMT activity is represented as a quantity relative to its maximum level. Thus,

$$e_{rel}=\frac{e}{e_{max}}$$ (8)

where *e* denotes TPMT enzyme activity. It is observed in clinical studies that the production of 6-TGN is negatively correlated with TPMT activity [31]. Hence, it is assumed that a fraction of 6-MP, proportional to (1 − *e*_*rel*_), is converted to 6-TGN. Consequently, the reaction rate is modeled as,

$$k_{cm}=k_{cm,max}\left(1-e_{rel}\right)$$ (9)

The description and units of all the state variables and parameters are listed in Table 1.

**Table 1:** Glossary of state variables and parameters for 6-MP model.

**Model variables**	**Description**	**Units**
*x*_*g*_	Amount of 6-MP in gut	pmol
*x*_*c*_	Amount of 6-MP in plasma	pmol
*x*_*m*_	Concentration of 6-TGN in RBCs	pmol/8x10^8^ RBCs
**Parameter**	**Description**	**Units**
*k*_*ab*_	Rate of absorption of 6-MP	per day
*k*_*el*_	Rate of elimination of 6-MP	per day
*k*_*cm*_	Rate of conversion of 6-MP to 6-TGN	pmol 6-MP converted/day
*K*	Michaelis–Menten constant	pmol
*k*_*me*_	Rate of elimination of 6-TGN	per day
*e*	Actual TPMT activity	Units per ml RBC
*e*_*max*_	Maximum TPMT activity	Units per ml RBC
*ν*_*cm*_	Stoichiometric coefficient for 6-TGN conversion	pmol 6-TGN produced per pmol 6-MP/8x10^8^ RBCs

### Bayesian Parameter Estimation: Off-line

#### Estimation of Individual Patients’ Parameter Distribution

The key step in the use of Bayesian population modeling for patient treatment individualization is the estimation of Bayesian posteriors for each patient using patient-specific data and a suitable prior. To accomplish this, first, parameter statistics are estimated to formulate a meaningful prior distribution for Bayesian calculations. The incorporation of prior knowledge is a key and unique aspect of the Bayesian framework. With large amount of data and well-defined parameters, the prior distribution may have a little impact on the posterior inference. However, when the data is far from adequate, such as in clinical settings, the prior distribution can play an important role, and hence it is expedient to devote considerable effort to obtain an informative prior. The model parameters for each of the individual patients who are part of the clinical study can be estimated using the maximum likelihood approach.

$$\underset{\theta }{max}\;L\left(D_{i}|\theta \right)$$ (10)

where $L\left(D_{i}|\theta ,\xi \right)$ is the likelihood function. With the assumption of experimental errors **ε** being independent and normally distributed, the likelihood function is given by,

$$L\left(D_{i}|\theta ,\xi \right)=\prod_{j\in \left\{1\ldots m\right\}}\left[\frac{1}{\sqrt{2\pi }\sigma_{j}}\text{exp}\left(-\frac{{\left(y_{ij}-{\hat{y}}_{ij}\right)}^{2}}{2\sigma_{j}^{2}}\right)\right]\;\forall i$$ (11)

where $D_{i}$ is the data set obtained from the *i* ^th^ patient.

Next, with the prior distribution formulated using this statistics, the posterior distribution can be calculated using Bayes theorem. According to Bayes theorem, the posterior distribution of the model parameters $p_{i}\left(\theta ,\xi |D_{i}\right)$ is given by Eq (12).

$$p_{i}\left(\theta ,\xi |D_{i}\right)=\frac{L\left(D_{i}|\theta ,\xi \right)\;p\left(\theta ,\xi \right)}{p\left(D_{i}\right)}$$ (12)

where *p*(**θ**, **ξ**) is the prior distribution. The denominator, $p\left(D_{i}\right)$, is the normalization factor equal to the expected value of the data irrespective of the parameters. The most common method for Bayesian inference is the Markov chain Monte Carlo (MCMC) sampling. However, MCMC can be very computationally intensive for large and complex models. An alternative methodology to MCMC sampling is the variational Bayes approximation. Variational Bayes translates the Bayesian inference into an optimization problem by approximating the posterior distribution using a known distribution form (e.g., a family of Gaussian distributions). The idea behind this methodology is that the logarithm of $p(D_{i})$ can be separated into two elements as shown in Eq (13) to (15).

$$\text{ln}\;p(D_{i})=L(q)+\text{KL}(q\|p)$$ (13)

$$L(q)=-\int_{\theta }\int_{\xi }\;q(\theta ,\xi |\phi )\;\text{ln}\left[\frac{q(\theta ,\xi |\phi )}{L(D_{i}|\theta ,\xi )\;p(\theta ,\xi )}\right]\;\text{d}\theta \text{d}\xi$$ (14)

$$\text{KL}(q\|p)=\int_{\theta }\int_{\xi }\;q(\theta ,\xi |\phi )\;\text{ln}\left[\frac{q(\theta ,\xi |\phi )}{p(\theta ,\xi |D_{i})}\right]\;\text{d}\theta \text{d}\xi$$ (15)

Here, *q*(**θ**, **ξ**|**ϕ**) is the parametric probability distribution that approximates the posterior distribution $p(\theta ,\xi |D_{i})$. ϕ is the set of parameters characterizing *q* (e.g., mean and covariance for a Gaussian distribution). If *q*(**θ**, **ξ**|**ϕ**) is free to be any probability function, then the maximum lower bound is obtained when the Kullback-Leibler (*KL*) divergence is zero. This occurs when *q*(**θ**, **ξ**|**ϕ**) exactly matches the posterior distribution [34]. Then, minimizing the *KL* divergence is equivalent to maximizing the lower bound of *L*. Therefore, the set of parameters Θ is determined as the one that maximizes *L*. Laínez et al. [35] developed a decomposition strategy to deal with the variational inference of models that are described by a set of DAE which will be followed for the variational Bayes approximation of the model for 6MP metabolism. Following this approach, the solution of the variational Bayes problem is decomposed into three steps: a maximum a posteriori optimization which is facilitated by using an orthogonal collocation approach, a preprocessing step which is based on the estimation of the eigenvectors of the posterior covariance matrix, and an expected propagation optimization problem [34]. The decomposition strategy has been implemented using the R [36] and GAMS [37] software packages. GAMS has been used for the optimization problems in the first and last steps.

#### Population Prior Distribution

One of the advantages of using the Bayesian approach is the systematic and prospective accumulation of information from each participating subject. Initially, patient-specific information from the retrospective clinical studies is accumulated to capture the population characteristics. This information is not only used for characterizing the new incoming patients but also constantly updated through the incorporation of new patients as part of the information. The individual posterior joint distributions obtained in the previous section are utilized to formulate an informative population prior distribution. A specific number of parameters (based on some weighting factor) from the converged posterior distribution are sampled to formulate the population prior distribution [24].

$$M\left(\theta ,\xi \right)=\sum_{i=1}^{N}w_{i}p_{i}\;\left(\theta ,\xi |D_{i}\right)$$ (16)

The weighting factor *w*_*i*_ is usually chosen based on the quality and quantity of data used to obtain these posterior distributions. Since the original data contained varied number of measurements, *w*_*i*_ is taken as a function of the number of data points for each patient.

### Global Sensitivity Analysis

The model parameters for a new patient cannot be estimated accurately unless measurements are made on that patient. Although the availability of an informative prior helps to reduce the number of measurements required, its impact can be considerably reduced when there are a number of model parameters to be estimated. However, if we can systematically identify and reduce the number of parameters which must be estimated a priori, it will aid in the efficient estimation of parameters online, when measurements are limited. In addition, it will significantly reduce the computation time which is also an essential part of timely decision making for a new patient in the clinical setting. The basis for reducing the number of parameters to be estimated stems from the observation of uncertainty in the dynamical systems. Uncertainty in the model output is primarily propagated from the uncertainty in the model input (i.e. parameters). Although it is true that the uncertainties in model parameters will have some effect on the model output, not all parameters have the same level of influence [38]. Consequently, it can be expected that the uncertainty in the estimation of the highly sensitive parameters will have the most significant impact on the model prediction. Thus, it becomes critical to estimate the most sensitive parameters as accurately as possible with the limited data available. Although less sensitive parameters have little effect on the measured variable, they impart indirect effect through other auxiliary variables and hence are fixed at a nominal value, instead of being eliminated or neglected. The error involved in such an approximation can be estimated as follows [39].

Let ${\hat{y}}_{i,0}$ represent ${\hat{y}}_{i}$ with $\theta_{i}={\left[\begin{array}{cc} {\hat{\theta }}_{i} & {\tilde{\theta }}_{i,0} \end{array}\right]}^{'}$ where ${\tilde{\theta }}_{i,0}$ denotes the insensitive parameters fixed at nominal value. For any parameter ${\tilde{\theta }}_{v},v=1,2,\ldots ,r$, if $S_{{\tilde{\theta }}_{v}}^{t}<<1$, then the error of approximating ${\hat{y}}_{i}$ with ${\hat{y}}_{i,0}$, $\delta \left({\tilde{\theta }}_{v,0}\right),$ by fixing ${\tilde{\theta }}_{v}$ at a nominal value ${\tilde{\theta }}_{v,0}$ can be estimated by

$$P\left[\delta \left({\tilde{\theta }}_{v,0}\right)<\left(1+\frac{1}{\varepsilon }\right)S_{{\tilde{\theta }}_{v}}^{t}\right]\geq 1-\varepsilon ;\;0<\varepsilon \leq 1$$ (17)

where $S_{{\tilde{\theta }}_{v}}^{t}$ is the total sensitivity of the model output corresponding to parameter ${\tilde{\theta }}_{v}$. For an arbitrarily small value of *ε* = 0.05, the probability of getting $\delta ({\tilde{\theta }}_{v,0})<21S_{{\tilde{\theta }}_{v}}^{t}$ is more than 0.95. Since physiological model parameters vary over a wide range, we used global sensitivity analysis (GSA) for estimating $S_{{\tilde{\theta }}_{v}}^{t}$. The Soboĺ method was used in this work to estimate the total sensitivity indices [39].

### Optimal Experimental Design

Clinical data, especially for new patients, are constrained due to economical and logistical reasons. Hence, we are interested in characterizing patients by measuring drug concentration with a minimum number of samples. By collecting samples at optimal time points, dictated by the design of experiment principles (DoE), one can estimate the model parameters accurately. Obviously, these optimal sampling points should be determined for the whole population with a detailed study on a class of patients with full set of data. Here, the population characteristics are enriched in M(θ,ξ) and will be used for sampling time determination. The average drug profile among patients studied is given by

EY(t)=∫M(θ,ξ)Y(x(t),θ,u)dθ (18)

Suppose we have a way to characterize the best choice of time at which these measurements are to be made, given the parameter vector **θ**. Denote this instant by T(θ). The average of this time is given by

ET=∫M(θ,ξ)T(θ)dθ (19)

Similarly, one could also consider T(Eθ) as a potential choice for the optimal time. To evaluate these measures, more generally, we may propose θ* such that the variance below is minimized.

$$\underset{\theta^{*}}{\text{min}}\;\int {\left[T\left(\theta^{*}\right)-T\left(\theta \right)\right]}^{2}\;M\left(\theta ,\xi \right)d\theta$$ (20)

The solution of the foregoing minimization problem will yield θ* and hence the best time for the measurement by evaluating $T\left(\theta^{*}\right)$. Eq (20) may be written further as

$$\underset{\theta^{*}}{\text{min}}\;\left[T^{2}\left(\theta^{*}\right)-2T\left(\theta^{*}\right)ET+VT+{\left(ET\right)}^{2}\right]=\underset{\theta^{*}}{\text{min}}\;\left[{\left(T\left(\theta^{*}\right)-ET\right)}^{2}+VT\right]$$ (21)

Clearly, Eq (21) is minimum when $T\left(\theta^{*}\right)=ET$. With this conclusion at hand, T(θ) is found using D-optimal criteria which attempts to minimize the volume of the hyper-ellipsoid spanned by the joint parameter space. This is achieved by maximizing the determinant of the Fisher information matrix as in Eq (22)

$$\underset{T\in T}{\text{max}}\;\underset{\theta \in \Theta }{\text{E}}\left\{\left|I(\theta ,\xi ,\varphi )\right|\right\}$$ (22)

In Eq 22, *φ* represents design parameters. The robust formulation is ensured by integrating the determinant over a representative population parameter space and weighted by the corresponding likelihood function.

$$\begin{array}{l} \underset{\theta \in \Theta }{\text{E}}\left\{\left|I(\theta ,\xi ,\varphi )\right|\right\}=\int_{\theta_{s}^{\text{min}}}^{\theta_{s}^{\text{max}}}\cdots \int_{\theta_{2}^{\text{min}}}^{\theta_{2}^{\text{max}}}\int_{\theta_{1}^{\text{min}}}^{\theta_{1}^{\text{max}}}\left|I\left(\theta ,\xi ,\varphi \right)\right|L\left(\theta ,\xi |D\right)d\theta_{1}d\theta_{2}\cdots d\theta_{s}\\ \;I\left(\theta ,\xi ,\varphi \right)=\sum_{i}^{l}\sum_{j}^{l}{\tilde{\sigma }}_{I}^{ij}S_{i}^{'}S_{j} \end{array}$$ (23)

where **S**_*i*_ is the sensitivity matrix defined as

$$S_{i}=\left[\begin{array}{ccc} {\frac{\partial {\hat{y}}_{i}}{\partial \theta_{1}}|}_{t_{1}} & \cdots & {\frac{\partial {\hat{y}}_{i}}{\partial \theta_{s}}|}_{t_{1}}\\ \vdots & \cdots & \vdots \\ {\frac{\partial {\hat{y}}_{i}}{\partial \theta_{1}}|}_{t_{m}} & \cdots & {\frac{\partial {\hat{y}}_{i}}{\partial \theta_{s}}|}_{t_{m}} \end{array}\right]$$ (24)

### Dose Optimization

With the availability of a patient-specific model, the final step in model-based individualized dosing is dose optimization to fulfill certain physiological objectives. The nature of the objective function selected is entirely dependent on the drug-disease-side effect combination. For 6-MP, the therapeutically effective range is defined in terms of 6-TGN concentration in peripheral RBCs. Moreover, the dosing strategy employed can depend on the degree to which the patient response can change over time. In some cases, the optimal dosing once determined can be repeated for a number of dosing cycles, unchanged [40]. In other situations, model mismatch and changes in patient response over time require repeated dose adjustments as patient response is tracked. This is the case in this work, thus, we utilize robust model predictive control (MPC) to optimize the dose due to its intrinsic capability to implement prediction and optimization under uncertainty [41–43]. In general, the MPC problem is formulated as solving the on-line finite-horizon, closed-loop optimal control problem subject to an underlying model and constraints involving state, input, and output. Based on the measurement obtained at time *τ*, the controller predicts the future moves of the system over a prediction horizon *T* and estimates the input that optimizes the predetermined open-loop performance objective function. To compensate for disturbances, model-patient mismatch and the *finite* nature of the optimization problem, only the first control action is implemented. The remaining samples are discarded and a new optimization problem is solved based on *Y*_*τ*+1_ at the next sampling step (*τ* + 1). The foregoing concept of MPC is analogous to the decision making process of a physician. When the patient arrives at the clinic for treatment, the physician diagnoses the patient, assesses the existing state of the disease, considers available treatment options, predicts the prognosis and administers the best available treatment to the patient but only until the next visit. When the patient returns for the next clinical visit, the physician repeats the same steps. Technically, MPC performs a similar task to what the physician does, perhaps in an optimal way, provided the model predictions are reasonable. While the traditional MPC algorithm is built with nominal dynamics of the model, we exploited the whole posterior distribution (with appropriate number of samples) of the individual patient parameters to account for the uncertainty.

Consider the equality constraint to maintain the drug concentration in the therapeutic level *TL*

$${\hat{y}}_{i}\left(u_{i}\left(\tau \right),x_{i}\left(\tau \right),\theta_{i},\xi_{i}\right)\in \left\{TL\right\},\;\tau =0,\ldots ,\text{T}$$ (25)

subject to the inequality constraints

$$g\left(u_{i}\left(\tau \right),x_{i}\left(\tau \right)\right)\leq 0,\;\tau =0,\ldots ,\text{T}$$ (26)

where T is the prediction horizon. *g* represent physiological constraints other than the clinical objective. For the case of regulating the system to the target concentration ℂ, the quadratic cost function is defined as follows.

$$J\left(U,\hat{y}\right)=\sum_{\tau }\int_{\theta }\int_{\xi }{\left(\mathbb{C}-\hat{y}\left(\theta_{i},\xi_{i},u_{i},\tau \right)\right)}^{2}\;L\left(\theta_{i},\xi_{i}|D_{i}\right)d\theta d\xi$$ (27)

where **U** = [*u*_0_,…, *u*_*T*−1_] is the optimization vector consisting of all the control inputs for *τ* = 0,…, T−1. The constrained finite time optimal control problem can be formulated as follows,

$$\underset{u}{\text{min}}\;J\left(U,\hat{y}\right)$$ (28)

subject to

$$\begin{array}{l} u_{\text{min}}\leq u(k)\leq u_{\text{max}}\\ \Delta u_{\text{min}}\leq u(k)-u(k-1)\leq \Delta u_{\text{max}} \end{array}$$ (29)

where *u*_*min*_ and *u*_*max*_ are minimum and maximum 6-MP doses allowed respectively. Δ*u*_*min*_ and Δ*u*_*max*_ signifies minimum and maximum allowed slew rate of 6-MP dose.

## Results

### Off-Line Population Model Building

**Fig 3:**
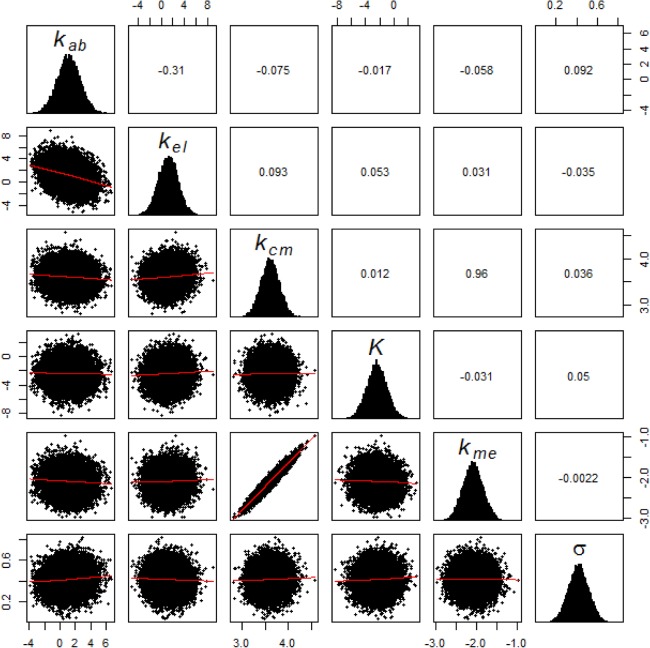
Marginal parameter distribution with correlation matrix estimated through Bayesian approach. The diagonal cells show the marginal distribution for individual parameters. The off-diagonal cells show the pairwise joint distribution of parameters and their corresponding correlation coefficient.

With the data sets *D1* and *D2*, model parameters were estimated using the maximum likelihood approach as explained in Section 2.4.1. Feasible ranges of parameters were chosen based on experimental and clinical studies from literature. The statistics of the estimated parameters are given in Table 2. We assumed that the priors, *p*(**θ**, **ξ**), are log-normally distributed with this statistics. Using this prior distribution and individual patients’ 6-TGN data $D_{i}$, the posterior joint distribution of model and error parameters were estimated for each patient through the variational-Bayes approach outlined earlier. The posterior distributions for selected patients were verified with MCMC approach as this is a standard approach for performing Bayesian estimations. MCMC was done using Metropolis-Hastings algorithm implemented through R package ‘mcmcpack’ [44,45]. The posterior parameter distribution is essentially an updated form of the prior distribution in light of new information i.e. individual patient’s 6-TGN concentration. Fig 3 shows marginal distribution of model and error parameters for a representative patient. Correlation coefficients for all parameters are acceptable except between *k*_*cm*_ and *k*_*me*_. To examine the adequacy of the model in representing drug concentration data for various patients, we employed the global lack-of-fit test described by Blau et al. [26]. This test compares the occurrence of the experimental points within the highest probability density (HPD) regions for concentration predicted by the model. By definition, HPD is the region in which there is a 100(1 − *α*)% probability that the true value falls within the area under $p\left(\hat{y}\left(t\right)\right)$ which satisfies $p\left(\hat{y}\left(t\right)\right)\geq p\left(y\left(t\right)\right)$. This confidence region (CR) is the smallest interval region among all credible intervals and hence is termed as the highest probability density region [46]. In our simulation, 259 out of 263 experimental points remained within the 95% HPD concentration confidence region, resulting in a confidence level for the lack-of-fit of 0.019. Since this value is less than 0.05, the selected model is adequate as measured by the global lack-of-fit test.

**Table 2:** Parameter statistics for prior distribution in Bayesian calculations.

Parameters	Mean	Covariance Matrix
*k*_*ab*_	*4.2*	$\left[\begin{array}{cccccc} & k_{ab} & k_{el} & k_{cm} & K & k_{me}\\ k_{ab} & 4.1 & & & & \\ k_{el} & 0.43 & 4.4 & & & \\ k_{cm} & 1.67 & 2.12 & 382 & & \\ K & 0.67 & -0.74 & 13.21 & 11.8 & \\ k_{me} & 0.003 & 0 & 0.059 & 0.42 & 0.3 \end{array}\right]$
*k*_*el*_	*3.8*	
*k*_*cm*_	*39.4*	
*K*	*15.11*	
*k*_*me*_	*0.08*	

**Fig 4:**
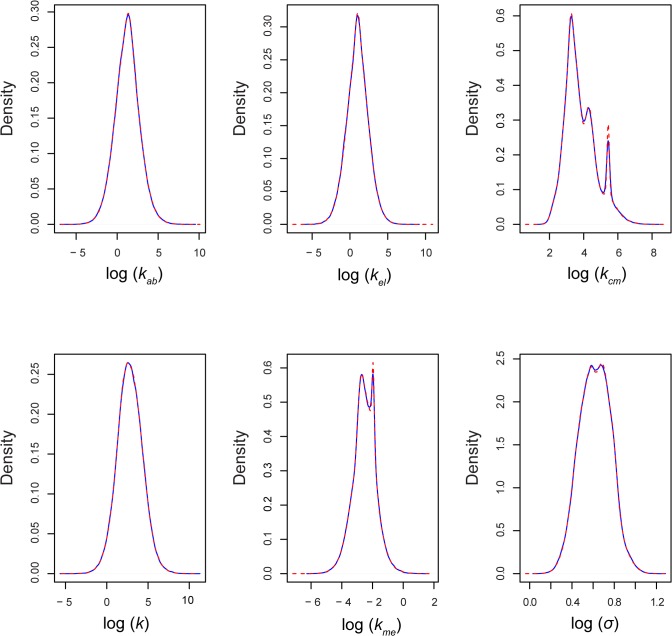
Population prior distribution for model and error parameters. Blue solid line indicates the actual distribution formulated by sampling individual patient distribution. The red dashed lines show the approximation by multivariate normal distribution.

**Fig 5:**
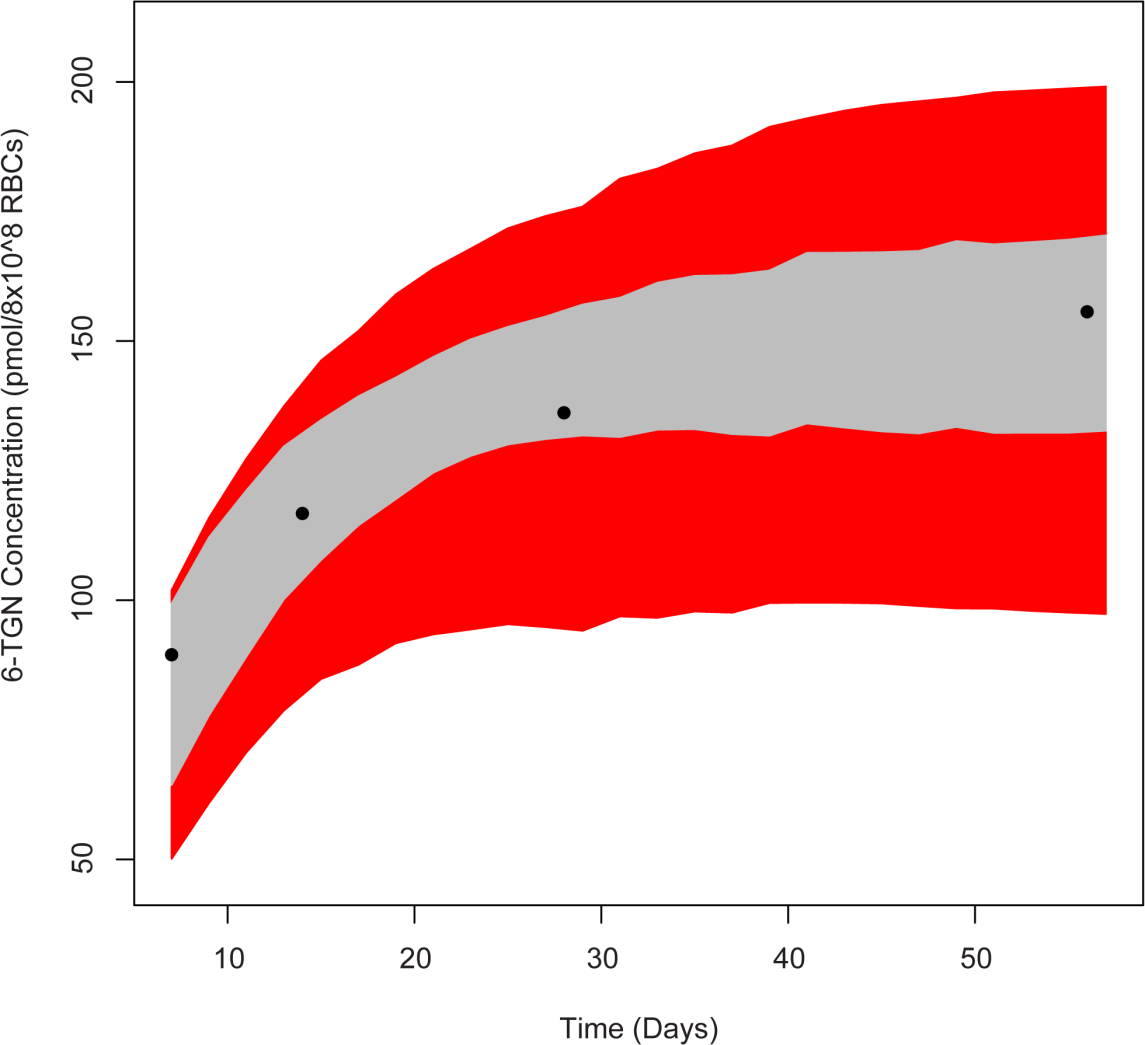
Comparison of concentration confidence region predicted using Bayesian approach for 6-MP model. The red region shows the prediction with non-informative prior. The gray region shows the improved CR with informative population prior formulated using several patient data. The solid dots represent the data collected from an individual patient.

Once the Bayesian parameter distribution is determined for all patients in the study, the population parameter distribution is formulated as explained in section 2.4.2. From each patient’s posterior parameter distribution, a specified number (10000*number of data points available for the corresponding patient) of samples are drawn. The final distribution for each parameter is approximated by a multivariate normal distribution. This approximate distribution is used as an informative population prior for all incoming new patients. In addition, every time a patient visits the clinic, this population prior is updated to include the new information. Fig 4 shows the population prior for model and error parameters. It is evident from the figure that most of the variability is explained by the reaction kinetic parameter *k*_*cm*_ and, to some extent by the elimination rate of 6-TGN *k*_*me*_. Fig 5 shows the comparison of 95% concentration confidence region estimated with a non-informative prior (i.e. formulated with MLE) and informative prior. The red region shows the 95% HPD when parameters are estimated with non-informative prior. As expected the confidence region obtained from informative prior (gray region) is narrower. This exemplifies the advantage of the Bayesian approach where accumulation of information results in improved accuracy of prediction.

### Model Reduction and Sampling Time Determination

Global sensitivity analysis was performed using the Sobol technique with 1000 set of parameters. Parameters were sampled through sparse grid sampling using statistics from the population prior distribution. Reaction and elimination rates of 6-TGN are the two parameters that emerged as highly sensitive ones. These two parameters have also displayed multimodal distributions in population priors (Fig 4). Similar observations were made in other population studies in literature [32]. Since the sensitivity indices varied with time, a cumulative error was calculated by assuming six representative time points across the treatment period. Accordingly, Eq (17) was modified as shown in Eq (30) with *ε* = 0.05.

$$P\left\{\delta \left(\theta_{i,0}\right)<\sum_{1}^{6}\left(1+\frac{1}{0.05}\right)S_{\theta_{i}}^{tot}\right\}\geq 0.95$$ (30)

**Fig 6:**
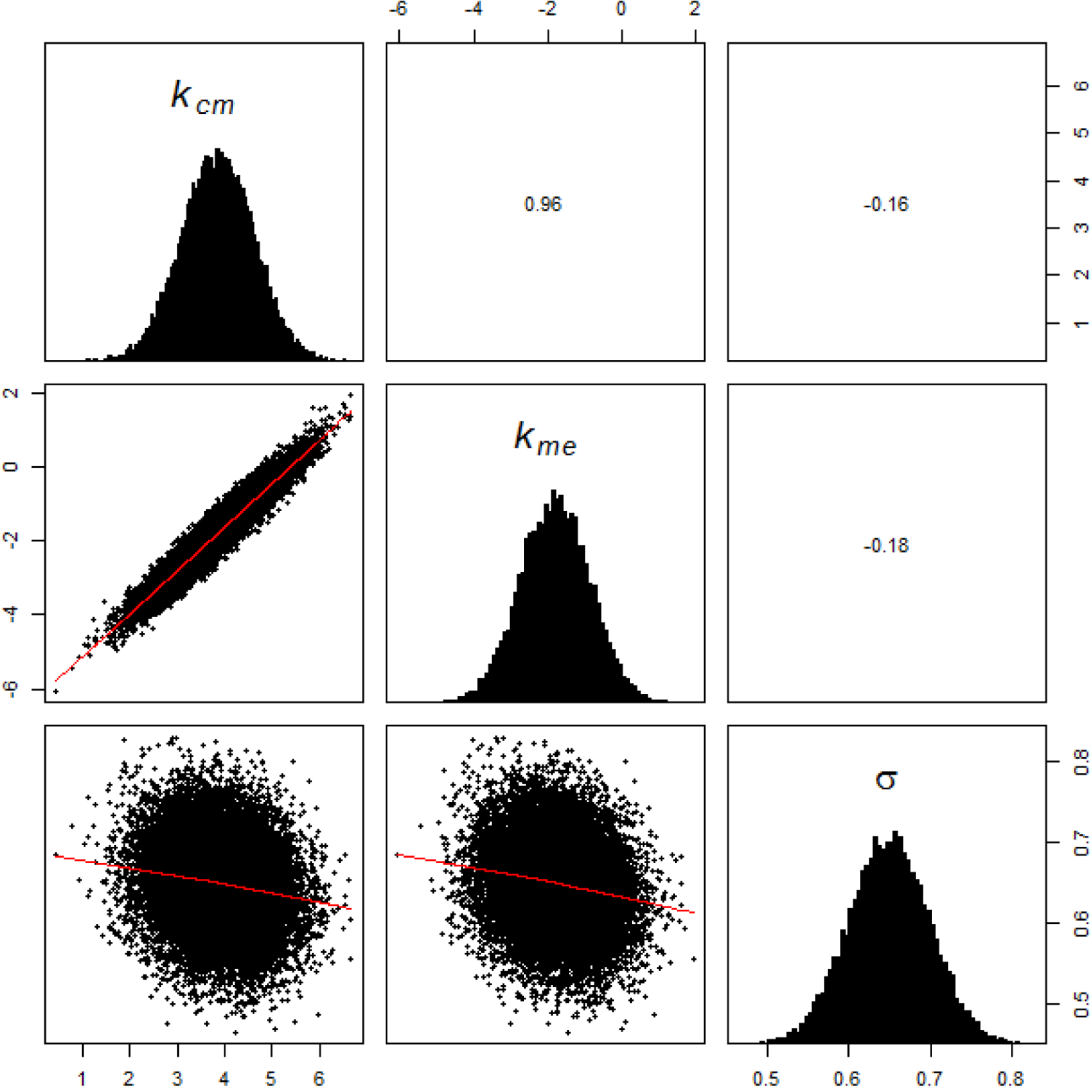
Marginal distribution of parameters showing correlation between *k*_*cm*_ and *k*_*me*_. Only *k*_*cm*_ and *k*_*me*_ are estimated, with all other parameters fixed at population mean, to reveal the correlation between them.

**Fig 7:**
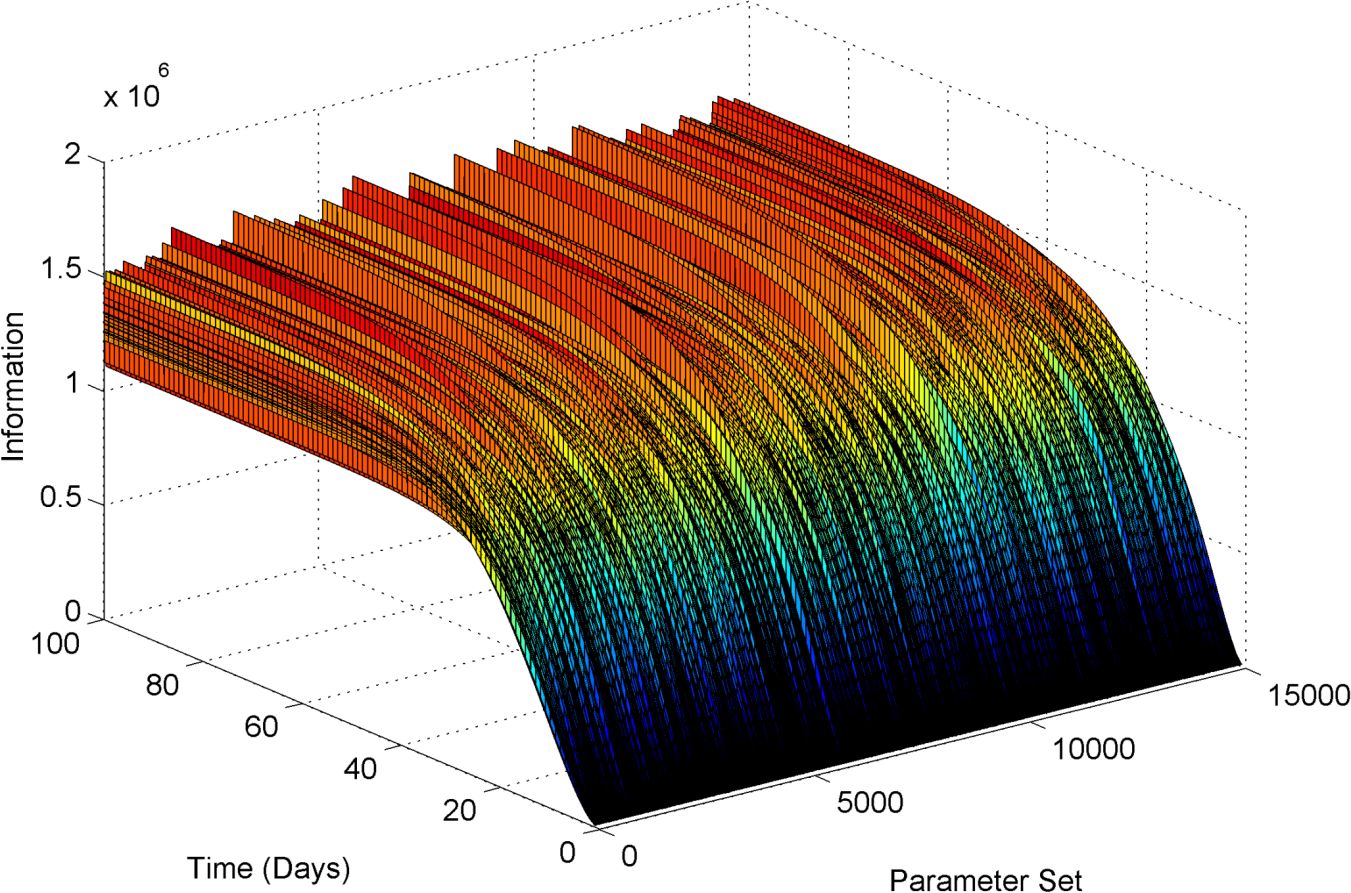
Evolution of information as a function of time and parameter set determined via optimal DoE technique.

Table 3 shows the error associated with all the parameters. Any parameter with less than 2% of the error associated with the most sensitive parameter will be regarded as less sensitive and hence fixed at the population mean for all individual patients. The most sensitive parameter and hence the highest error involved was found to be *k*_*cm*_. Taking this as the reference error, the error involved was less than 2% for all parameters, except *k*_*me*_. However, as mentioned before, the correlation between *k*_*cm*_ and *k*_*me*_ is 0.96. Bayesian estimation with only *k*_*cm*_ and *k*_*me*_ as estimable parameters (other parameters were fixed at population mean) confirmed this trend and is shown in Fig 6. As a result, it suffices to estimate only *k*_*cm*_ and fix all other parameters at the population mean for new patients. Consequently, the experimental design was formulated with the objective of improving the precision of parameter estimation for *k*_*cm*_. Fig 7 shows the evolution of Fisher’s information as a function of time and parameter sets. From the figure, the maximum information is made available towards the steady state of the model. Concentration densities simulated with population prior also pointed that the maximum variation in the concentration distribution resulted when the drug concentrations are higher. However, it is not prudent to wait until the steady state to gather the data and identify the new patient. Hence, by compromising about 5% of the maximum information, 35^th^ day was determined as the optimal time to collect blood sample to measure the 6-TGN concentration.

**Table 3:** List of parameters identified for deriving patient-specific model (bold face) together with other fixed parameters (regular face). Cumulative error, calculated at 1, 10, 20, 50, 75 and 100 days according to Eq 30, is given in column 2. % error is given in column 3.

6-MP Model (Variable for GSA: *x*_*m*_)		
Parameters	$P\left\{\delta ({\tilde{\theta }}_{i,0})<(.)\right\}\geq 0.95$	% error(actual/maximum)
***k***_***cm***_	**88.57**	**100**
***k***_***me***_	**47.84**	**54.01**
*K*	1.34	1.51
*k*_*el*_	0.025	0.028
*k*_*ab*_	8.7x10^-4^	9.8x10^-4^

### Online Implementation

To this stage, we accumulated the information available in the literature by formulating a population prior distribution and showed the strategy for estimating the parameter for a new patient with a minimum number of samples. Since the optimal sampling time falls on the 35^th^ day from the beginning of treatment, it puts the patient in the dark for this period as there is no patient-specific model available for dose optimization. To overcome this drawback, we employed TPMT activity as an additional piece of information available up in the biological chain-of-response. For this purpose, data *D3*, consisting of TPMT measurement and corresponding 6-TGN concentration, are utilized to predict the optimum dose during the first 35 days. TPMT activity in these patients ranged from 7- to 30 U/ml/hr. With an activity window of 2 U/ml/hr. (i.e. Group 1: 7.01–9.00 U/ml/hr., Group 2: 9.01–11.00 U/ml/hr. etc.), 11 patient groups are formed based on their TPMT enzyme activity. Using the population prior distribution generated in section 3.1, a new set of parameter distributions were obtained for *k*_*cm*_ for all patients in *D3*. With these distributions, group prior distributions were formulated as follows,

$$M_{G}\left(\theta ,\xi \right)=\sum_{i=1}^{N_{g}}w_{i}p_{i}\left(\theta ,\xi |D_{i}\right)\;\forall G,\;G=1,2,\ldots ,11$$ (31)

where $M_{G}\left(\theta ,\xi \right)$ is the prior distribution for the group defined by the TPMT enzyme activity and *N*_*g*_ is the number of patients in each activity group that ranged from 3–27. Eq. 31 is equivalent to Eq 16 in that Eq 31 considers the subset of patients having similar gene expression pattern as the population instead of the whole population. In summary, when a patient arrives at the clinic (at time *t* = 0), TPMT enzyme activity is measured and the patient will be placed in one of the 11 groups. Dosing decisions until *t* = 35 days are made with the assumption that the patient behaves as an ‘average’ of this enzyme activity group and hence dose optimization is performed with $M_{G}\left(\theta ,\xi \right)$ relevant for this patient. Once 6-TGN concentration is measured on the 35^th^ day, $M_{G}\left(\theta ,\xi \right)$ is updated for this patient and the dose optimization is performed with the patient-specific model.

### Evaluation of Uncertainty

**Fig 8:**
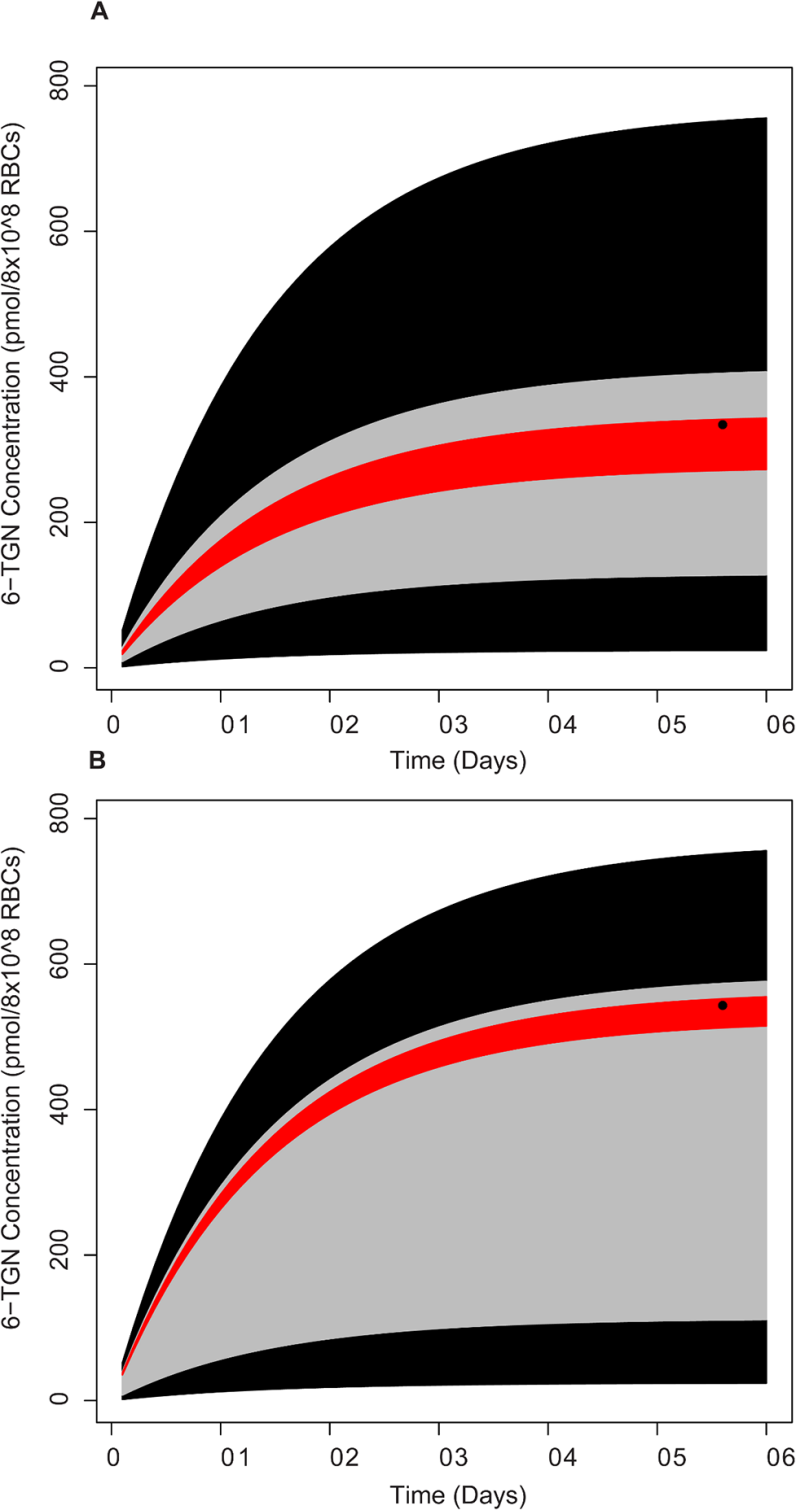
Comparison of 95% CR predicted using different information on biological chain-of-response for two representative patients. The black region represents the population. The gray region shows the prediction when only TPMT enzyme activity is measured. The red region shows the 95% CR predicted when just one measurement of 6-TGN is available (the solid dot).

Another important objective of this work is to evaluate the impact of measuring various entities in biological chain-of-response on the uncertainty in predicting variables used for therapeutic drug monitoring and optimization. Despite the attractiveness of the DNA sequencing and gene expression profiling in characterizing patients, the more downstream variables, such as enzyme activity and active metabolite concentration, provide a much more robust indication of underlying response and thus helping to minimize the uncertainty. For this purpose, the individual parameter distributions are sampled to represent various measurements viz. whole population, groups based on TPMT genotype, groups based on TPMT enzyme activity and individual patient 6-TGN concentration measured at a single time point. Two TPMT genotype groups were formed based on enzyme activity. A TPMT enzyme activity of less than 10 U/ml/hr. is deemed as *TPMT* ^*H*^ */TPMT* ^*L*^ (heterozygous) and an activity of more than 10 U/ml/hr. is deemed as *TPMT* ^*H*^ */TPMT* ^*H*^ (homozygous-High) [31]. There are no *TPMT* ^*L*^ */TPMT* ^*L*^ (homozygous-Low) patients found in this study. As Fig 8 shows, the black region is the 95% CR of concentration for the whole population included in this study. Without any attempt to collect genetic/phenotypic measurements from a patient, the most that can be aspired for is that the concentration will fall in the range of 50–650 pmol/8x10^8^ RBCs. This is indeed a reflection of the current status of clinical practice. Obviously, such uncertainties would preclude the possibility of dose individualization. The confidence regions for both the TPMT genotype groups were no different from this population CR. The gray region shows the 95% CR for the group having a TPMT activity of 15–17 U/ml/hr. With the measurement of a slightly downstream marker, the CR is narrower compared to the CR predicted for the population. However, when the 6-TGN concentration is measured and the model is adapted subsequently, the 95% CR is much narrower with notably lower uncertainty. With this patient-specific model, making accurate prediction of drug concentration at hand, one can venture into dose optimization strategies to direct the drug concentration into a desired region that maximizes the efficacy and minimizes the side-effects.

### Dose Optimization

**Fig 9:**
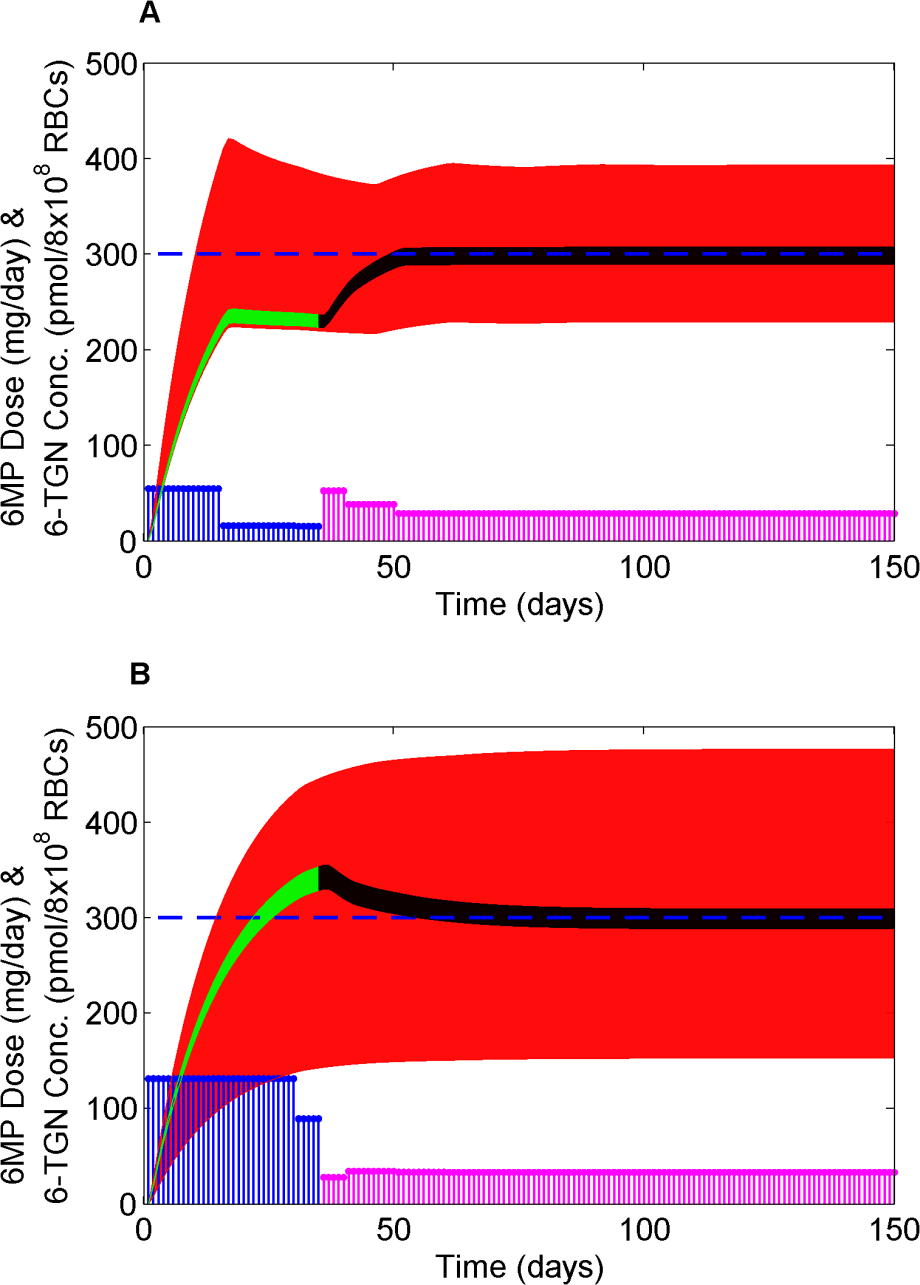
Optimal 6-MP dosing and corresponding optimal 6-TGN concentration profile for two representative patients. The red region is the 95% CR of concentration optimized for the group. Green region is 95% CR back calculated until the 6-TGN measurement is taken. Black region is the optimized profile with patient-specific model after 6-TGN measurement on the 35^th^ day. Blue and pink stems represent 6-MP doses before and after 6-TGN measurement respectively. The blue dashed line designates the concentration target. See text for details.

Dose optimization is performed using the robust MPC strategy to achieve a therapeutically effective 6-TGN concentration. All dosing calculations are based on 15 days sampling horizon with 75 days treatment window as patients visit the clinic every two weeks. 50 parameter sets are sampled either from the group prior or patient-specific distribution. The combined error for all the parameters, weighted by their corresponding likelihood, was minimized within the MPC optimization. Clinical studies recommend a therapeutic 6-TGN concentration of 235–400 pmol/8x10^8^ RBCs for an effective management of both efficacy and toxicity [47,48]. Hence, we optimized 6-MP input with a target 6-TGN concentration of 300 pmol/8x10^8^ RBCs. Fig 9 shows the optimal 6-MP input together with resultant 6-TGN concentration for patients who had different response in relation to their respective groups. As mentioned earlier, without the patient-specific model until day 35, the dose is optimized based on the enzyme activity. The red region shows the 95% CR of concentration optimized for an enzyme activity group. When the patient-specific model is obtained with a measurement on the 35^th^ day, the optimized region shifts based on the drug concentration measured and reaches the target with narrow CR (shown in black). The CR in green shows the back calculation with the same dose as that of group optimum but with patient-specific model. The patient in subplot A had a lower reaction rate in relation to the group to which he/she belonged and hence when the actual measurement becomes available the dose had to be increased to push the 6-TGN concentration higher. The opposite is true for the patient in subplot B. Dose inputs for different patients suggest that the dosage varied as much as 200% and as low as 25% of the standard dose, which is not uncommon in the clinical practice. Although there are obvious and significant differences between the standard and optimized 6-MP usage, the merit of dose optimization should be viewed from the maximization of therapeutic benefits rather than the reduction of drug input as the cost of drug is only a fraction of the overall healthcare spending.

## Discussion

Individualized treatment strategies are increasingly favored and extensively recommended for many drugs, especially for deadly diseases like cancer. Availability of modern analytical facilities and computing power for *in-silico* approaches support recommendations to further such efforts. Implementation of these advances in clinics faces several challenges. Among these, the most important issues are the technical ones which are mainly concerned with robust determination of optimal dosing for an individual patient.

In this work, we have engineered a strategy for a model-based individualized treatment for an important chemotherapeutic drug. We have devised a Bayesian approach to describe the population characteristics and subsequently utilized it for robust estimation of patient-specific parameters in light of sparse clinical data. The Bayesian approach is inherently suitable to consolidate the information from various sources in various formats into a prior distribution. Highly informative priors play a significant role in dealing with situations where real-time decisions have to be made with partial information i.e. sparse data. The use of distributions of parameters arising from the Bayesian approach, instead of parameter point estimates, enabled us to perform a robust estimation of sampling times, to optimize the dose and to quantify the associated uncertainty. More importantly, we have shown the advantage of measuring the downstream biomolecular phenotypic indicators, rather than an upstream genotypic marker, in minimizing the uncertainty in predicting clinical variables of our interest. While the latest DNA microarray technologies have made genotyping an inexpensive way to characterize the patients, its utility in individualized dosing comes at the price of significant uncertainty.

In section 1.3, we described how important it is to measure the downstream clinical variables for accurate prediction of dose response. Although 6-TGN concentration itself was shown to be a valuable indicator of clinical efficacy and toxicity and proven useful in certain clinical conditions where efficacy/toxicity measures are categorical and highly subjective, extension to include cellular response will certainly add value to the approach. Additional variations while the active drug imparts cytotoxicity on various cell populations may play a role and impose another level of uncertainty. Hence, an interesting and important extension to this work would be to connect this model to relevant pharmacodynamic variables (cellular response). With regard to 6-MP treatment, these cellular responses primarily involve the bone marrow cell population. Works are in progress to consider these extensions.

Besides the issue of ‘technical know-how’, clinical implementation is also constrained by physiological, logistical, economic and social factors. Physiological issues are concerned with whether the technical solutions are realizable within the physiological constraints. For example, if the approach demands several additional blood samples, it will be prohibitive to implement in pediatric patients, no matter how beneficial it is. Certain types of measurements, such as those from the bone marrow are highly restricted. Logistical issues surface mainly during the translational phase. They arise due to incompatible resources at the healthcare facilities. For example, if the algorithm requires a measurement 6 hours post-dose, managing patients arriving at different times in the clinics (inpatient or outpatient) would be an issue. In addition, timely analysis of samples and reporting the results would be important consideration. The economic issues are obviously concerned with the additional cost involved for procedures necessitated by the individualized approach. Individualized treatment means clinically identifying or characterizing an individual patient through genotyping and/or phenotyping that invariably requires additional testing/ lab assays. Basically, one has to show the potential benefits, both short- and long-term, weighed over the cost, to convince third party payers to cover the tests. The final and important social issues encompass communication, information sharing, education to healthcare providers and patients on the new approach, patient compliance, policy, ethical and privacy issues. It has been our aim in this work to address some of the technical issues within the physiological constraints. We believe that by comprehensively addressing the foregoing two issues and corroborating the potential incentives will pave way for overcoming the rest. This will ensure that the patients are treated with the suitable dose, dictated by their own genetic/phenotypic background, ultimately resulting in improved quality-of-life.
